# Case of Congenital Defect of the Rectum

**Published:** 1864-03

**Authors:** B. W. Robinson

**Affiliations:** Fayetteville, N. C.


**Art. VII.**—*Case of Congenital Defect of Rectum* Reported by B. W. Robinson, M. D., Fayetteville, N. C.

An infant, aged seven and a half months, just returned from a long journey, came under my professional care for slight hemorrhage from the bowels. *Embonpoint* good. Slight catarrhal affection, and more or less of dysuria noticed from birth. Had a fortnight before a dysenteric attack attended with severe tenesmus, which seemed to have yielded readily. Has had within a few days dejections of blood; teething.

Inspection discovered a slight fissure in verge of anus, anteriorly. A few days after, perceptible fullness of perineum, which resulted in abscess and discharged itself by a longitudinal slit of four or five lines, very close to, and on left of the raphe. In a few days more, a parallel opening of about equal extent occured on the right. Subsequently, after an interval of two or three days, urine flowed per rectum. Up to this time excretion from the bladder had been through the natural channel. Paroxysms of severe suffering prompted an examination, cautiously made, with the little finger, which detected marked fullness and fluctuation in the prostate gland. This discharged in a day or two, and then a rent of four to six lines occurred in upper portion of the scrotum, through which fæces were extruded. Successive acts of defecation increased the rents (those on either side of the raphe being thrown into one,) 'till a frightful fissure, laying open rectum to the extent of two inches—membranous portion of urethra, and nearly the entire scrotum along the mesial line, was induced.

Duration of the case, about seven weeks. Alimentation had been for the most part good. Bowels acting generally three or four times in twenty-four hours. Alvine evacutions healthy. Dentition going on, during which four incisors were evolved. Occasionally, febrile disturbance. Catarrhal symptoms were troublesome latterly. Hemorrhage of only occasional occurrance—at one time exceeding a tablespoonful, and rarely more than a teaspoonful.

After a night of sleeplessness and suffering, patient sank suddenly. Death by asthenia.

*Necroscopy.*—Notable emaciation of lower extremities.—Small purulent deposit in sub-pubal areolar tissue. Abdominal viscera healthy. Slight adhesion of pelvic organs. Bladder empty—contracted to a containing capacity of not more than two fluid drachms. Rent through rectum (1½ to 2 inches in extent,) and membrano-prostatic portion of urethra—save these, no evidence of disease. Rigid scrutiny completely dissipated a diagnostic hypothesis which assumed the presence of, and causation by, a foreign body.

*Deduction.*—Congenital defect—want of integrity of structure in mesial line, which allowed disparting, during tenesmus of original attack, and subsequent defecatory efforts.

